# Interstitial lung disease: a review of classification, etiology, epidemiology, clinical diagnosis, pharmacological and non-pharmacological treatment

**DOI:** 10.3389/fmed.2024.1296890

**Published:** 2024-04-18

**Authors:** Malik A. Althobiani, Anne-Marie Russell, Joseph Jacob, Yatharth Ranjan, Amos A. Folarin, John R. Hurst, Joanna C. Porter

**Affiliations:** ^1^Royal Free Campus, UCL Respiratory, University College London, London, United Kingdom; ^2^Department of Respiratory Therapy, Faculty of Medical Rehabilitation Sciences, King Abdulaziz University, Jeddah, Saudi Arabia; ^3^School of Health and Care Professions, University of Exeter, Exeter, United Kingdom; ^4^School of Medicine and Health, University of Birmingham, Birmingham, United Kingdom; ^5^UCL Respiratory, University College London, London, United Kingdom; ^6^Satsuma Lab, Centre for Medical Image Computing, University College London Respiratory, University College London, London, United Kingdom; ^7^Institute of Psychiatry, Psychology and Neuroscience, King's College London, London, United Kingdom; ^8^NIHR Biomedical Research Centre at South London and Maudsley NHS Foundation Trust, King's College London, London, United Kingdom; ^9^Institute of Health Informatics, University College London, London, United Kingdom; ^10^NIHR Biomedical Research Centre at University College London Hospitals, NHS Foundation Trust, London, United Kingdom

**Keywords:** interstitial lung disease, idiopathic pulmonary fibrosis, sarcoidosis, hypersensitivity pneumonitis, nonspecific interstitial pneumonia

## Abstract

Interstitial lung diseases (ILDs) refer to a heterogeneous and complex group of conditions characterized by inflammation, fibrosis, or both, in the interstitium of the lungs. This results in impaired gas exchange, leading to a worsening of respiratory symptoms and a decline in lung function. While the etiology of some ILDs is unclear, most cases can be traced back to factors such as genetic predispositions, environmental exposures (including allergens, toxins, and air pollution), underlying autoimmune diseases, or the use of certain medications. There has been an increase in research and evidence aimed at identifying etiology, understanding epidemiology, improving clinical diagnosis, and developing both pharmacological and non-pharmacological treatments. This review provides a comprehensive overview of the current state of knowledge in the field of interstitial lung diseases.

## Interstitial lung disease

**Figure 1:**
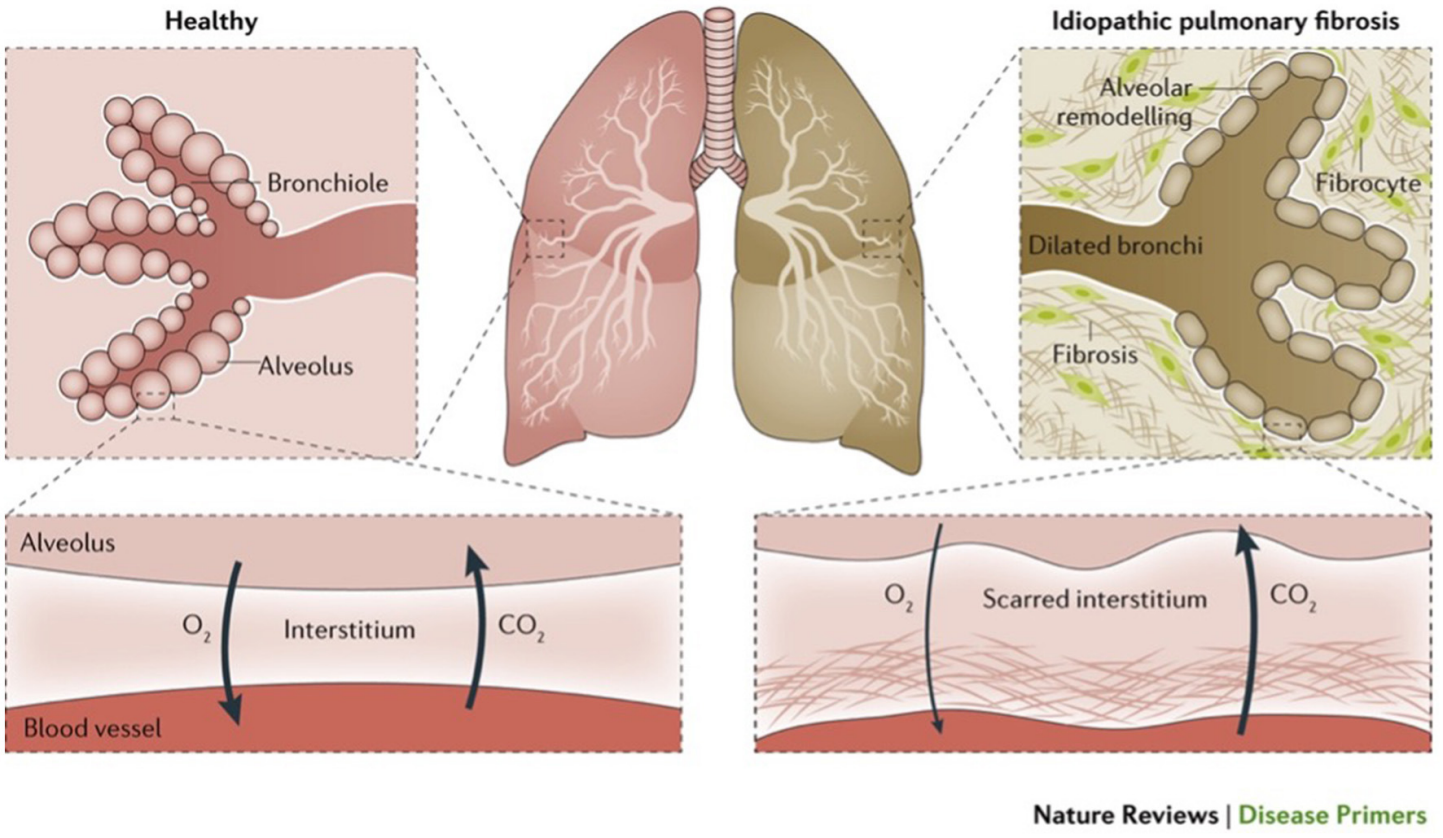
Illustrates normal lungs and lungs with a type of ILD (idiopathic pulmonary fibrosis) (1).

Interstitial lung disease (ILD) is an umbrella term for ~200 different diseases that may result in inflammation and scarring of the lung tissue (Figure 1) (2). ILD is characterized by progressive dyspnoea, cough, hypoxia, impaired lung function, diffuse bilateral infiltrates on imaging, inflammation, fibrosis, limited patient mobility and reduced quality-of-life (QOL) (3). Most ILD cases result from an etiological factor, such as exposure to allergens, hazardous material, asbestos, drugs or an underlying autoimmune disease (2–5). The development of these cases is a complex process that is influenced by a variety of factors, including the individual's genetic traits, and exposure to environmental pollutants (6). Idiopathic Pulmonary Fibrosis is the most aggressive form of ILD, causing progressive and permanent lung scarring. It causes a chronic and irreversible lung disease with a poor prognostic outcome with a median survival rate of 3–5 years post-diagnosis if left untreated (5, 7, 8). Although two antifibrotic medications demonstrated a significant reduction in the rate of disease progression, it remains difficult to predict disease behavior for individual patients (5, 7, 8). The purpose of this review is to provide up-to-date information on interstitial lung disease (ILD), with a particular emphasis on definition, classifications, etiology, epidemiology, diagnosis, pharmacological, and non-pharmacological management.

**Figure 2:**
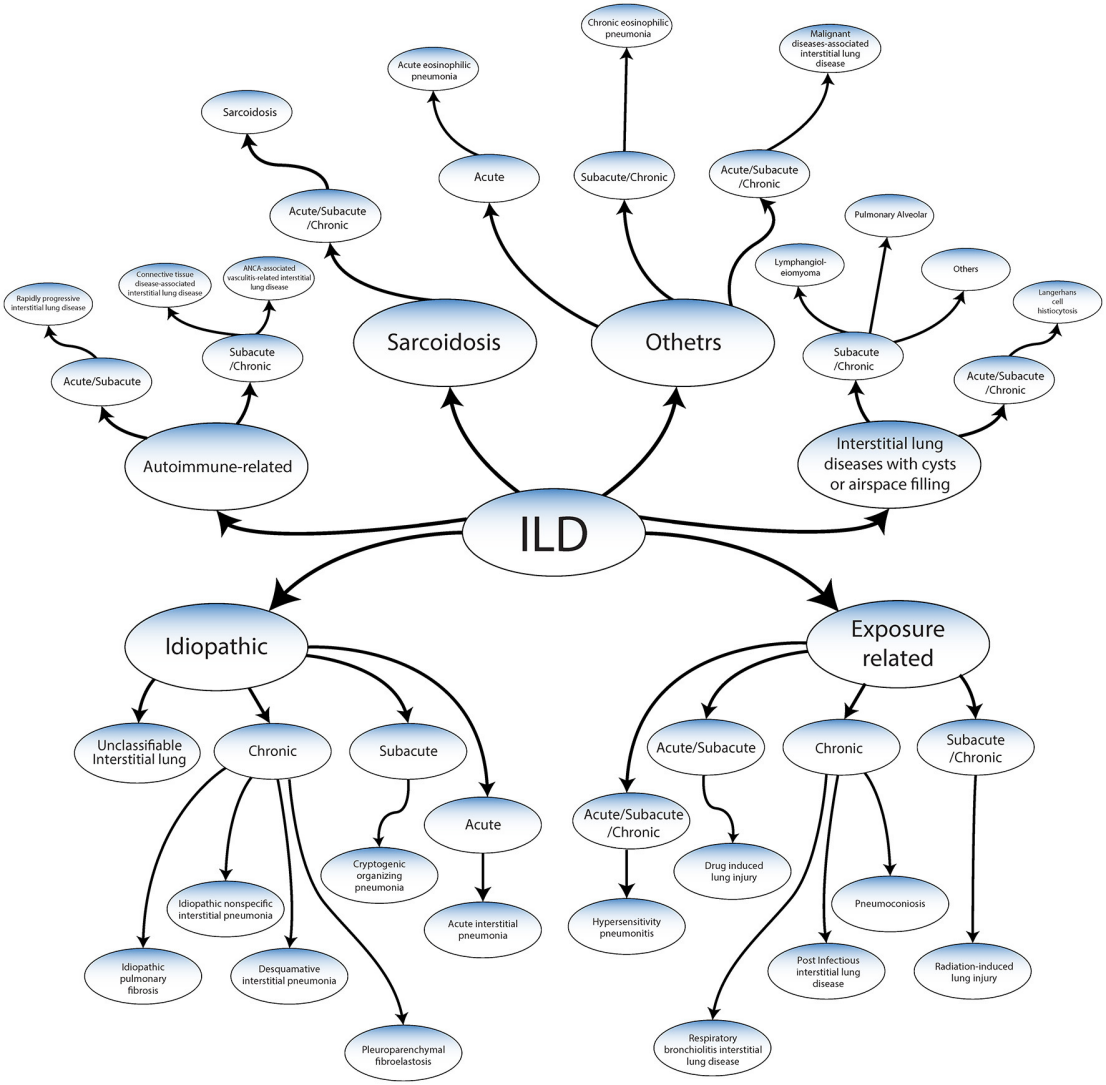
Classification of ILDs adapted from ATS/ERS 2013 guidelines and Wijsenbeek et al. (9).

ILD has been classified into the following categories based on a recent publication by the American Thoracic Society Consensus Statements (Figure 2) (2–5, 10–12).

## Classification

### Idiopathic

Idiopathic interstitial pneumonias (IIPs), represent a broad spectrum of diseases, each demonstrating distinct clinical features and rates of progression (3, 6). These diseases can be classified into several types, including:

➢ Idiopathic pulmonary fibrosis which is chronic and has a high risk of developing a progressive-fibrosing phenotype.
➢ Chronic IIPs: these can further be divided into various forms such as desquamative interstitial pneumonia, or pleuroparenchymal fibroelastosis, idiopathic nonspecific interstitial pneumonia.
➢ Acute IIPs: a prime example of this category is acute interstitial pneumonia.
➢ Subacute IIPs: an example of this type is a cryptogenic organizing pneumonia.
➢ Unclassifiable interstitial lung disease: these are IPPs that cannot be accurately categorized due to their unique and mixed characteristics.

The cause of these idiopathic conditions remains elusive, but their progression tends to be characterized by persistent and often worsening fibrosis, functional lung impairment, and a declining prognosis (13). The most severe and common idiopathic form is idiopathic pulmonary fibrosis (IPF), accounting for ~50% of all reported IIPs for which there is no cure (2–5, 11, 14). The considerable variability seen among patients makes individual outcome prediction difficult (3–5). Some IPF patients experience rapid progression and others experience slow progression. A systematic review suggested that gastro esophageal reflux disease (GERD) is associated with IPF (15). There is also a higher incidence among individuals who actively smoking cigarettes at the time of diagnosis or have a history of smoking (2, 13). Pharmacological treatments for IPF have historically included triple therapy [prednisolone, azathioprine, and N-acetyl cysteine (NAC)] and NAC monotherapy which were proven to be ineffective (16–19). The last decade has seen introduction and widespread use of two anifibrotic agents (Pirfenidone and Nintedanib) which have been shown to slow the rate of forced vital capacity decline (16–19).

### Autoimmune-related

➢ Acute conditions, rapid progressive interstitial lung disease such as diffuse alveolar hemorrhage in ANCA-associated vasculitis or in systemic lupus erythematosus and anti-MDA5-antibody-associated amyopathic dermatomyositis.
➢ Chronic disease includes connective tissue disease-associated interstitial lung disease (CTD-ILD). Diseases such as systemic sclerosis and rheumatoid arthritis are CTD-ILD conditions that can cause inflammation and damage to various body tissues, including the lungs (20). CTDs are also associated with other less common types such as idiopathic inflammatory myopathy (dermatomyositis, polymyositis), systemic lupus erythematosus, and Sjögren's syndrome (21). Connective tissue disease is systemic immune diseases where T- and B-cells attack the lung tissue, causing inflammation. Although specific causes are unknown, environmental factors, toxins and genetics contribute to CTD-ILD (21, 22). ILD is prevalent in an estimated 15% of patients with CTDs, and slightly higher in patients with SSc and RA. The risk of CTD-ILDs is associated with women younger than 50 years of age (21) and other comorbidities (23).

### Exposure-related ILD diseases

Some exposure-related diseases are chronic and have a high risk of developing a progressive-fibrosing phenotype, such as hypersensitivity pneumonitis, which is mostly related to the inhalation of organic particles (e.g., domestic, or occupational exposure to mold, birds or other exposures).

➢ Chronic such as pneumoconiosis due to inhalation of inorganic substances, respiratory bronchiolitis-interstitial lung disease and postinfectious interstitial lung disease. Environmental and occupational exposures represent a significant cause of certain ILDs (24). Long-term exposure to airborne irritants such as silica dust, asbestos fibers, and specific animal droppings can lead to inflammation and fibrosis in the lung tissue, resulting in conditions such as silicosis, asbestosis, or hypersensitivity pneumonitis. These harmful substances can cause direct damage to the lungs, triggering an inflammatory response that can lead to fibrotic scarring (20, 24).
➢ Acute, such as drug or radiation-induced lung injury, may occur due to chemotherapy drugs, antibiotics, anti-inflammatory medications, and certain heart drugs can cause lung tissue damage, leading to drug-induced ILDs. The mechanism may involve direct toxicity to lung tissue, allergic reaction, or initiation of an autoimmune response (25).

### Interstitial lung diseases with cysts or airspace filling

➢ Langerhans cell histiocytosis.
➢ Lymphangioleiomyomatosis (LAM).
➢ Pulmonary alveolar proteinosis.

Lymphangioleiomyomatosis which is characterized by abnormal growth of smooth muscle cells primarily in women of childbearing age (26, 27). Pulmonary Langerhans cell histiocytosis, which involves accumulation of a specific type of immune cell in the lungs (27). Pulmonary alveolar proteinosis and pulmonary alveolar microlithiasis, both characterized by abnormal accumulation of substances in the alveoli.

### ILDs related to distinct primary diseases

These are more likely to show a progressive-fibrosing phenotype such as in sarcoidosis. Sarcoidosis is a multisystem chronic inflammatory disease that affects multiple organs in the body, including lymph nodes, eyes, skin, and, most commonly (28), the lungs. It leads to abnormal macrophages and T-lymphocyte activation that targets body organs (29). It was first described in 1889 by Besnier (30) and characterized by the non-malignant formation of non-caseating epithelioid granulomas in the pulmonary alveolus. The most commonly reported symptoms include cough, fever, breathlessness, night sweats, weight loss and bilateral hilar lymphadenopathy. The causes are unknown, but a growing body of research has investigated the mechanism of granuloma formation, including genetic and environmental factors (31). The most commonly reported genetic factor linked to sarcoidosis is HLA-DRBI (32, 33). The role of interactions at the major histocompatibility complex (MHC) binding site is also significant (34). Environmental factors, including exposure to insecticides, clay, pine tree pollen, talc, zirconium, and aluminum, can influence both the prognosis and susceptibility of sarcoidosis (35). Sarcoidosis is more common in African Americans with a reported incident rate of 17–35 per 100,000 population and in people aged between 20 and 39 years (32, 36). Severity of the disease is also greater in black populations (37). Hispanics and Asians have been reported to have the lowest incidence rate of 1–3 per 100,00 population. Diagnosing sarcoidosis depends on clinical, radiological, and histological evidence of the formation of non-caseating granulomas (38). Phenotypic impairments in pulmonary function vary by race, gender, disease duration, and tobacco use (39). The differential diagnoses include viral and bacterial infections, autoimmune disease, hematological malignancy, and mycobacterium infection (38, 40). Although flexible bronchoscopy has demonstrated accurate results for sarcoidosis diagnosis, endobronchial ultrasound-guided transbronchial needle aspiration is the recommended sampling method in patients suspected of having sarcoidosis (41, 42).

### Other ILD diseases

Examples include chronic eosinophilic pneumonia, malignant diseases with associated interstitial lung disease (e.g., lymphangitis carcinomatosis), and acute eosinophilic pneumonia (43).

## Etiology

While the pathophysiological mechanisms are not entirely understood, ILD is categorized as a restrictive lung disease that reduces lung expansion and total lung capacity (TLC) (44). It causes scars and damage to alveoli leading to changes in lung function and decreased lung capacity and gas exchange (1). Scarring and lung damage are linked to the creation of fibroblastic foci, where fibroblasts proliferate in response to alveolar cell injury. Triggered by transforming growth factor beta (TGF-β), this process transforms fibroblasts into myofibroblasts, which secrete collagen, leading to fibrosis (45). Understanding the functionality of the lung and how ILD specifically changes lung structure and function helps to differentiate it from other lung diseases (2). Although the impact of socioeconomic factors on clinical outcomes in patients with interstitial lung disease is not well characterized, they may also play a role (46). A range of causes contribute to the development of ILD. These causes can be categorized into known and unknown origin (3).

### Inflammation

Several factors can trigger inflammation in interstitial lung disease, with autoimmune disease being the most common. Autoimmune disease (AD) is defined as “a clinical syndrome caused by the activation of T cells or B cells, or both, in the absence of an ongoing infection or other discernible cause” (47). Previous research has identified differences in the blood cells' genetic expression between healthy individuals and those with fibrotic lung diseases, suggesting that blood markers could help in understanding these conditions better (48). Monocytes have been correlated with disease severity in IPF and have been associated with abnormalities in various blood cell types, including macrophages and lymphocytes. In the single-cell resolution analysis of immune cells in IPF and FHP, it was found that there are common changes in monocyte populations across both IPF and FHP, while lymphocytes exhibit disease-specific differences (48). The inflammatory response is a complex cascade of events involving various immune cells, such as lymphocytes and macrophages, as well as the release of inflammatory mediators like cytokines and chemokines. This response is usually beneficial for dealing with acute injuries or infections (10). However, in the case of interstitial lung disease (ILD), the inflammatory process becomes dysregulated and can lead to progressive lung damage (9). In addition, the role of Autotaxin (ATX), an enzyme responsible for producing lysophosphatidic acid (LPA), significantly contributes to the inflammation and fibrosis observed in interstitial lung diseases (49). The persistent inflammation leads to further tissue damage, creating a vicious cycle. Abnormal multiplication and excessive protein secretion by fibroblasts, including collagen, contribute to the process of fibrosis (10). These proteins accumulate and harden, leading to fibrosis, which stiffens the lung tissue and impairs its ability to exchange oxygen and carbon dioxide effectively (10). Scarring and lung damage are linked to the creation of fibroblastic foci, where fibroblasts proliferate in response to alveolar cell injury. Triggered by transforming growth factor beta (TGF-β), this process transforms fibroblasts into myofibroblasts, which secrete collagen, leading to fibrosis (50).

### Risk factors

Recent advancements in single-cell RNA sequencing (scRNA-seq) technologies have provided novel insights into the pathogenesis of IPF (51). Study by Unterman et al. (51) has revealed reveals that certain immune aberrations, like the increase in classical monocytes, are observed in both stable and progressive forms of IPF. However, significant distinctions are evident, particularly regarding regulatory T cells (Tregs). An increase in Tregs, alongside the formation of a specific lung-blood immune recruitment axis, is more prominently associated with or specific to progressive IPF (51).

### Smoking

Cigarette smoking is a notable contributing cause of ILD and a significant contributor to numerous diseases and deaths (52). Cigarettes contain an estimated 5,000 chemicals (53), most of which have been contributed to deaths and a large number of diseases (54, 55). Smoking has been associated with a million deaths in the previous century, and could result in over 1 billion deaths in the current century (56). These include acute eosinophilic pneumonia and pulmonary hemorrhage syndrome, where smoking plays a crucial role in their pathogenesis. Among chronic lung diseases, desquamative interstitial pneumonia (DIP), respiratory bronchiolitis associated interstitial lung disease (RB-ILD), pulmonary Langerhans cell granulomatosis (PLCH) demonstrated associated with smoking (57). Despite the clear linkage between smoking and these diseases, the relationship with other lung conditions like Idiopathic Pulmonary Fibrosis (IPF) and Rheumatoid Arthritis-related ILD (RA-ILD) is more complex. While there is a high prevalence of smoking amongst patients with these conditions, smoking has not been definitively established as their cause. Interestingly, conditions such as sarcoidosis and hypersensitivity pneumonitis show no etiological association with smoking, despite similar prevalence rates (58).

#### Desquamative interstitial pneumonia

Desquamative interstitial pneumonia (DIP) was first described in 1965 by Liebow et al. (59) and was later described as alveolar macrophage pneumonia due to its nature of diffuse filling of alveolar spaces (3). The most commonly presented symptoms are dyspnoea and cough. Less common symptoms are fever, chest pain, and fatigue (60). Radiographs typically show fine reticulation and bilateral ground-glass opacities in the basal part of the lung, widespread build-up of pigmented macrophages within the alveoli, an increase in numbers of type II alveolar epithelial cells and commonly diffuse thickening of alveolar septa, septal fibrosis and mild interstitial inflammation (60).

DIP is a restrictive disease that results in a reduced diffusing capacity of carbon monoxide, and it can be accurately diagnosed with a surgical lung biopsy. Although mostly found among smokers (60), 20% of DIP cases are associated with non-smokers and linked to etiological causes, including environmental exposures (molds, dust), occupational exposures to inorganic particles (solder fumes, flock-workers, diesel fumes, tungsten carbide), viral infections (hepatitis C virus, cytomegalovirus), medications (sirolimus, nitrofurantoin), and connective tissue disease (rheumatoid arthritis, systemic sclerosis, rarely systemic erythematosus lupus) (57).

Treatment should initial focus on removing the exposure, such as smoking cessation, in conjunction with medical intervention (60). A recent systematic review by Hellemons et al. found that corticosteroids, specifically prednisolone, were used to treat 91% of patients; with less commonly used treatments being primarily methylprednisolone and antibiotics such as clarithromycin, azathioprine, ribavirin, chloroquine and cyclophosphamide (60).

#### Respiratory bronchiolitis-associated interstitial lung disease

Respiratory bronchiolitis associated interstitial lung disease (RB-ILD) is a type of interstitial pneumonia commonly linked to smoking; and it is associated with accumulation of pigmented macrophages in the peribronchiolar alveolar spaces and distal bronchioles. Respiratory bronchiolitis is characterized by shortness of breath (61).

### Air pollution

The air we inhale contains 78% nitrogen, 21% oxygen, 0.9% argon, 0.04% carbon dioxide, and other trace gases (62). However, it also carries pollutants, oxidants, and other harmful particles that damage the tiny thin alveolar epithelium, which leads to abnormal lung functionality. Inhaling these toxins trigger an immune system response which, as a result, can lead to a variety of different disorders (63).

Several studies have established air pollution as a risk factor for the development and progression of ILD (64, 65). A study conducted in Northern Italy demonstrated that an increase in nitrogen dioxide (NO_2_) exposure during the cold season was associated with an increased incidence of IPF (66). Another study in India revealed a significant correlation between increased city-wide concentrations of particulate matter with a diameter ≤2.5 μm (PM2.5) and the percentage of hypersensitivity pneumonitis cases registered in that center's ILD registry (67). A study of 25 patients with IPF using weekly spirometry found that higher weekly mean concentrations of nitrogen dioxide (NO_2_), particulate matter 2.5 (PM2.5), and particulate matter 10 (PM10) contributed to lower mean forced vital (64) capacities (FVCs) over the study period (68, 69).

### Occupational exposure

Occupational exposure to certain substances has been shown to contribute to various types of ILD (70, 71), although the majority of occupational exposure takes time to show symptoms for example, coal mine dust, asbestos fibers, crystalline silica, mold, and cobalt-tungsten carbide alloy are commonly reported as the causes of ILD. Occupational exposure to asbestos, silica, and coal dust was estimated to have caused 125,000 deaths according to the Global Burden of Disease Study in 2010 (72). The prevalence of this disease has been increasing among workers exposed to occupational dust. In the Jiangsu Province of China, for example, 9,243 cases were reported from 2006 to 2017, and in the United Kingdom, the most common type of pneumoconiosis is asbestosis (73). Furthermore, a systematic review demonstrated that gases, fumes, and vapors (71) may account for 26% of IPF (71).

### Genetic

A growing body of evidence shows that genetic factors play a role in some of ILD onset (74, 75). It is now understood that certain inherited traits, such as rare pathogenic variants in genes associated with telomere maintenance and surfactant production (76), common single nucleotide polymorphisms (77), and reduced leukocyte telomere length can contribute to an increased risk of ILD (78). An estimated fifth of IPF cases can be traced back to familial origins, leading to the term familial pulmonary fibrosis when two or more blood relatives are diagnosed with ILD (79, 80).

Understanding these genetic variants has the potential to aid predicting the prognosis of ILD. Specifically, single nucleotide polymorphisms (SNPs), which are single base pair changes in the DNA sequence are among the DNA variations polymorphisms can affect the function of a gene and the activity of the encoded protein (81). In patients IPF, MUC5B promoter polymorphism (rs35705950) MUC5B, which is a mucin protein, has been linked to an increased risk of developing the disease (82). In particular, IPF susceptibility has been linked to polymorphisms in the promoter of the gene encoding salivary mucin, 5b (MUC5B), and the Toll-interacting protein (rs5743890; TOLLIP). These genetic variants, despite their association with a fairly mild phenotype, serve as promising targets for precision medicine in IPF (83).

### Age and gender

Recent research highlights the importance of considering both gender and age in understanding the epidemiology, pathophysiology, and treatment responses of ILDs (26, 84). For instance, studies on non-IPF ILDs, such as hypersensitivity pneumonitis and connective tissue disease-associated ILDs, indicate gender and age-related differences in incidence, disease progression, and outcomes (85).

Male sex and older age are associated with a higher incidence rate of IPF (5). Specifically, the risk of developing IPF is 1.5–2 times more in men than women (86). This is because telomeres shorten with age, and IPF is associated with telomere shortening. It has been observed that the risk of developing hypersensitivity pneumonitis is more pronounced in men than in women (87). The development of interstitial lung disease linked to connective tissue disease (CTD-ILD) is more commonly observed in younger women who are nonsmokers (88). The prevalence of systemic lupus erythematosus is significantly higher in women than in men, with the ratio of affected individuals ranging from 7:1 to 10:1 (89). This disparity is also evident in other forms of interstitial lung disease, such as lymphangioleiomyomatosis (LAM), which primarily affects white women (26).

Similar trends are observed in other ILDs, indicating a potential underlying biological mechanism linked to that influences susceptibility to lung fibrosis (85, 89). Research suggests that hormonal differences, especially the protective role of estrogen against lung tissue damage, may contribute to these disparities. Moreover, lifestyle, including smoking habits which are historically more prevalent among men, could further exacerbate the risk of developing ILDs (89, 90).

Aging is a risk factor for ILDs, with a significant increase in incidence observed among older adults. This trend is particularly evident in IPF, where the disease predominantly affects individuals over the age of 65 (26). The association between aging and ILDs can be partially attributed to the biological process of telomere shortening (75, 78). Telomeres, protective caps at the end of chromosomes, naturally shorten as individuals age, contributing to cellular senescence and reduced regenerative capacity (75, 78). In IPF and possibly other ILDs, accelerated telomere shortening due to genetic factors or environmental exposures can lead to premature lung aging and increased vulnerability to fibrotic processes. Studies have found that men over 65 exhibit the most significant telomere shortening, correlating with the highest risk for developing IPF (75, 78). However, it's essential to consider that aging affects lung physiology and immune responses in both genders, contributing to a general increase in ILD susceptibility among older adult (91).

## Epidemiology

ILD is a growing global health issue, with an increase of 86% in ILD-related mortality (92, 93) and predicted to account for 0.26% of all-cause mortality in 2027 in the world A recent study found that the prevalence of ILD ranged from 6.3 to 71 per 100,000 people, and the incidence rate of ILD ranged from 1 to 31.5 per 100,000 people per year (94). Another study from France reported a prevalence rate of 97.9 per 100,000 and incidence of 19.4 per 100,000 for individual per year (95). Specifically, this study reported Sarcoidosis and IPF as the most commonly reported ILDs in Europe and North America (93). In addition, hypersensitivity pneumonitis was commonly reported condition in Asia, with reported rates of ranging from 10.7 to 47.3% of all ILDs in India and 12.6% in Pakistan. On the other hand, connective tissue disease-associated interstitial lung disease (CTD-ILD) was commonly reported in Canada (33.3%). In Belgium, the incidence of CTD-ILD was estimated to be around 7.5% cases per 100,000 population per year (96). The British Lung Foundation estimated the incidence rate of IPF is about 50 per 100,000 population per year in the UK (97). There are limited studies regarding the prevalence and incidence rate of ILD in Saudi Arabia. However, a recently, a study by Kaul et al. (93) reported variability in the global prevalence of ILD. This epidemiologic study presented findings on the prevalence of connective tissue disease cases in Saudi Arabia, reporting a rate of (34.8%) (93). In addition, one study by Alhamad (98) recruited 330 patients with ILD from a single tertiary care center. Included patients were native Saudis with a mean age of 55.4 years and were mainly female (61.2%). As above, 34.8% of cases of ILD were CTD-ILD, followed by IPF (23.3%), Sarcoidosis (20%), and HP (6.3%) (98).

## Diagnosis

### Importance of accurate diagnosis in ILD

**Figure 3:**
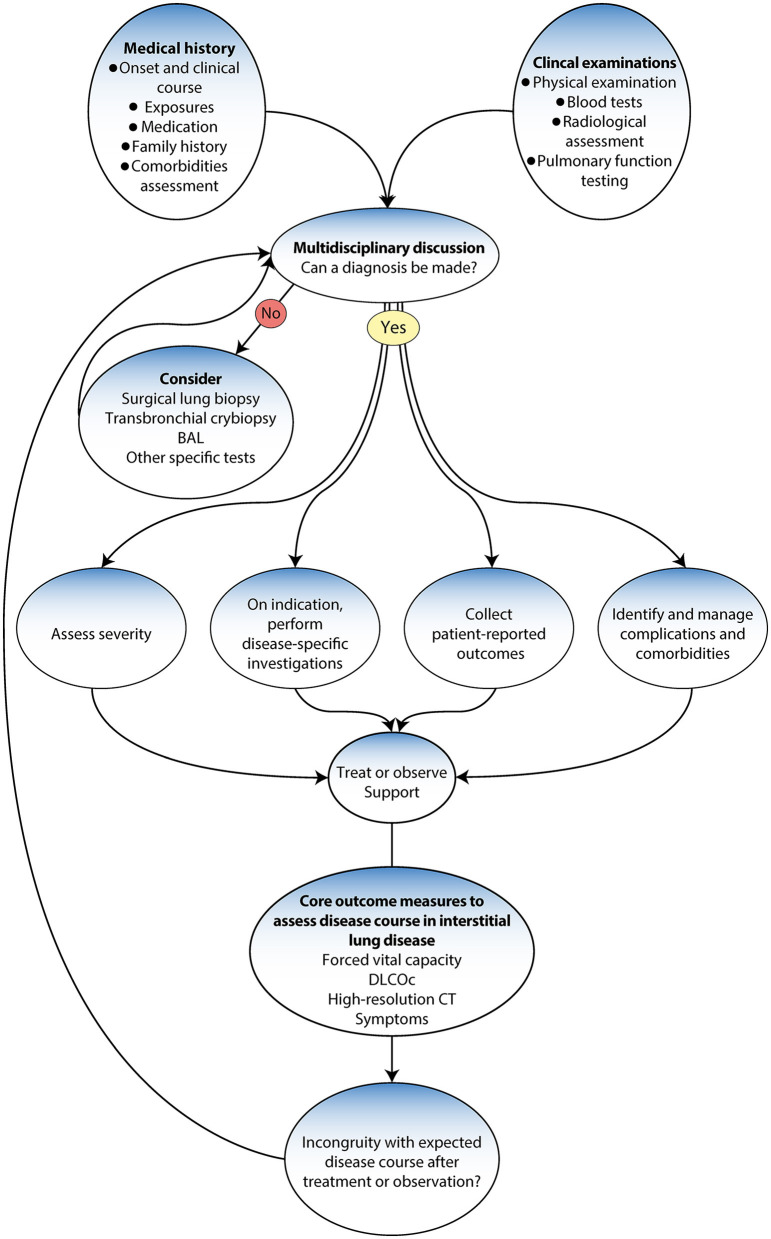
Diagnostic algorithm adapted from Wijsenbeek et al. (9).

Accurate diagnosis is essential to ensuring the most favorable disease prognosis for patients (99). Thus, following the international guidelines and the evidence-based diagnostic pathways may save lives (5). The updated guidelines have changed how ILD is diagnosed, from depending primarily on a histopathological investigation to a new gold standard multidisciplinary team (MDT) approach that uses various methods and tools (4, 5, 9). Previous studies have demonstrated that a multidisciplinary approach enhances the accuracy in the final decision among ILD specialists (100, 101) (Figure 3).

### Composition and process of MDT

Conducting a thorough investigation and detailed assessment that takes into account various factors is essential to facilitate these processes (5, 100). These include a comprehensive investigation and a detailed assessment of medical history, onset and clinical course, family history, occupational and environmental exposure, toxic response, medications (chemotherapy) exposure, and comorbidities (10, 101). It also includes physical examinations, blood tests, pulmonary function tests and radiological assessments (101). High-resolution chest computed tomography (HRCT) can help identify any structural abnormalities or interstitial lung disease that may contribute to the patient's symptoms (5). It is also paramount to ensure the MDT is available for reassessment of treatment when the patient is not following the anticipated disease course (9). If no specific interstitial disease diagnosis is made, a more invasive investigation may be considered, such as bronchoscopy, bronchoalveolar lavage, transbronchial lung cryobiopsy (BLC), or surgical lung biopsy (5, 11). If the ILD does not fit a pattern or is a rare type, such as lymphangioleiomyomatosis (LAM) or Langerhans cell histiocytosis (LCH), the case is referred to a specialist center, where the condition is reviewed using advanced diagnostic methods and tools (10, 102). Furthermore, advancements in genetic testing, serum biomarkers, and artificial intelligence technologies for radiologic and histopathological assessment are revolutionizing the diagnostic approach (103).

### Factors to consider in diagnosis

Once a diagnosis is made, the next step involves assessing the severity of the disease, typically by disease-specific investigations (104). These tests help identify the ILD subtype and its associated severity. Patient-reported outcomes are also collected to understand the impacts of the disease from the patient's perspective (105). These insights offer a holistic understanding of the patient's condition and guide subsequent management strategy steps (99). In addition to these measures, identifying and managing complications and comorbidities are crucial (105). This multidisciplinary approach guarantees that not only is the primary ILD treated but also that other existing conditions or complications are addressed; hence minimizing the potential negative impacts of any comorbidities on the patient's overall health status. The MDT has a responsibility to refer to the wider interdisciplinary team to ensure holistic care and follow up (5).

### Disease-specific investigations

The monitoring of core outcome measures like forced vital capacity, diffusing capacity of the lung for carbon monoxide, chest radiographs and high-resolution CT scans enables the tracking of the course and progression of the disease (99). Also monitoring symptoms that are most commonly reported, such as breathlessness, cough, and fatigue, which cause physical and emotional distress and, in addition, low quality of life (5, 106).

### Importance of identifying and addressing comorbidities

Interstitial lung diseases are commonly associated with several comorbidities influencing their course, progression, and overall patient outcomes (107). These comorbidities include both acute and chronic infections, gastro-oesophageal reflux, pulmonary hypertension, cardiac disease, pulmonary embolism, lung cancer, obstructive sleep apnoea, and depression (108). Gastro-oesophageal reflux is observed in up to 94% of IPF patients. Pulmonary hypertension is also common, with prevalence rates ranging from 32 to 85% in IPF, 5%−74% in Sarcoidosis, and 5%−12% in systemic sclerosis (SSc). Cardiac diseases are found in about 60% of IPF and 20% of sarcoidosis cases. Lung cancer is seen in ~4.4%−10% of IPF cases. Obstructive sleep apnoea is highly prevalent in ILDs, with rates of 60%−90% in IPF, 50% in SSc-ILD, and 65% in Sarcoidosis. Depression is also a common comorbidity in ILDs, with a prevalence >20% in ILDs overall and 11%−50% in IPF.

For patients diagnosed with definite or probable idiopathic pulmonary fibrosis (IPF), the patient may be suitable for antifibrotic treatment or considered for clinical trials. In these cases, the specialist center reviews the condition and recommends suitable treatment paths. The shared care approach is again employed to ensure ongoing monitoring and management (5, 9).

### Evaluating disease severity and risk-benefit profiles

In patients with a definite or possible diagnosis of nonspecific interstitial pneumonitis (NSIP) or connective tissue disease (CTD) or if further diagnostic review is necessary—which may include a biopsy—the specialist center's MDT takes the lead (24). After the review, the specialist center provides feedback, and the suggested course of action is initiated at the local level. Regular reviews at the specialist center ensure ongoing patient monitoring and care plan adaptability. Ongoing monitoring helps identify any inconsistency in the expected disease course after treatment or observation and allows for necessary adjustments in the management plan. Throughout this process, the key role of multidisciplinary discussion is reiterated, ensuring a comprehensive, patient-centerd approach to managing ILD (101).

### Pulmonary function testing

**Figure 4:**
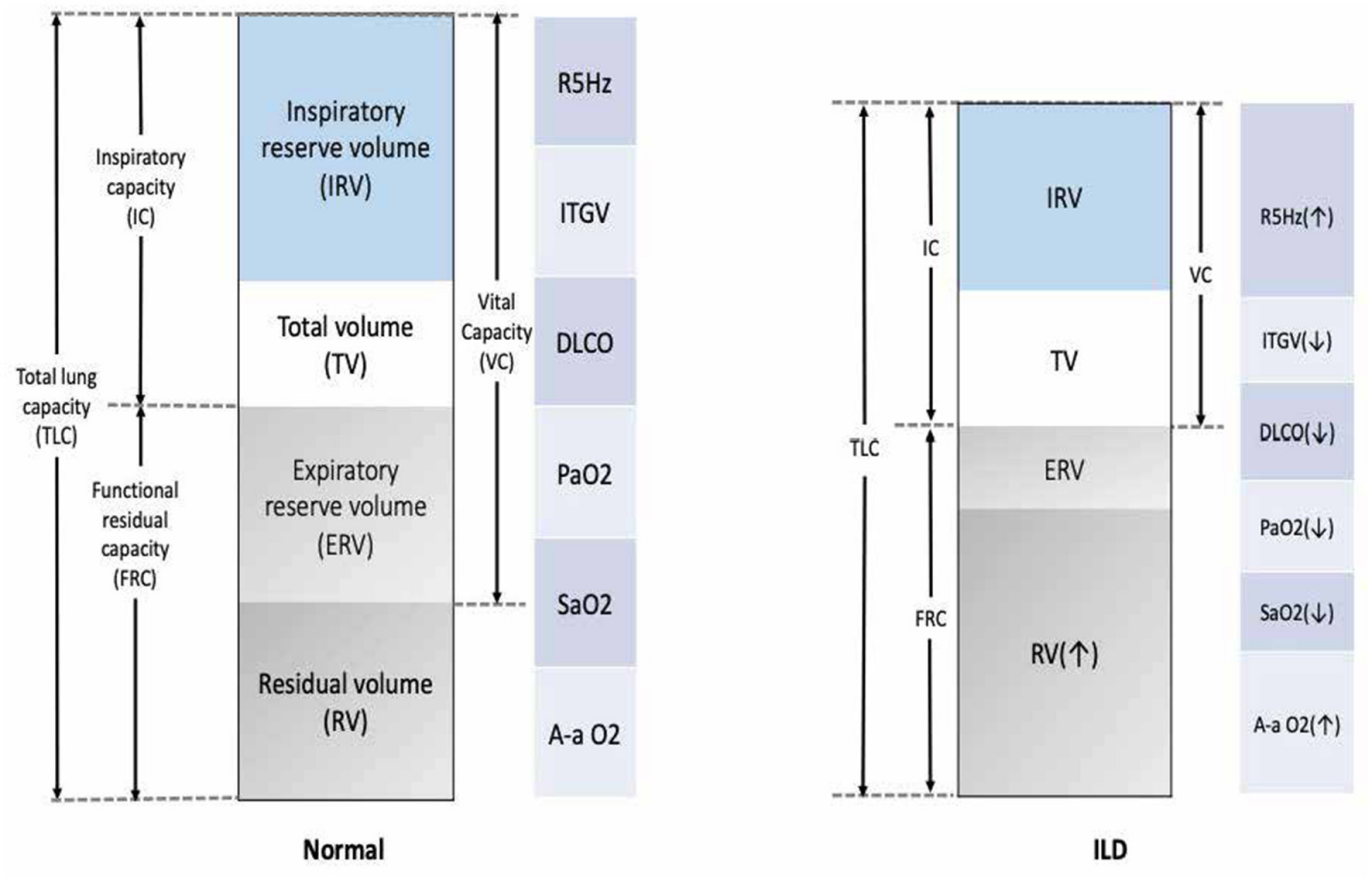
Common functional abnormalities in patients with ILD. VC, vital capacity; FRC, functional residual capacity; ITGV, intrathoracic gas volume; TLC, total lung capacity; R5Hz, resistance measured at 5 Hertz; DLCO, diffusion capacity; PaO_2_, partial pressure for oxygen; SaO_2_, arterial saturation of oxygen; A-a O_2_ gradient, alveolar-arterial oxygen gradient; VO_2_, oxygen uptake.

A lung function test is crucial for diagnosing obstructive vs. restrictive lung function. ILD is a restrictive disease that shows a low total lung capacity, residual volume, and lower forced vital capacity and causes a low (DLCO; Figure 4) (109). Spirometer is regularly used in patients with ILD to assess treatment response and monitor disease progression (5). The gold standard for assessing the clinical endpoint in IPF has been hospital-based spirometer measurements of forced vital capacity (FVC) every 3 months. Unfortunately, obtaining lung function tests can be challenging, and delays are common. For example, it was not possible to conduct lung function tests during the COVID-19 pandemic lockdown, and many patients with ILD were unable to monitor their conditions (110, 111). Moreover, the cost and burden on the national healthcare system further add to the difficulties faced in obtaining these essential tests (4).

### Radiological investigation and other specialist investigations

**Figure 5:**
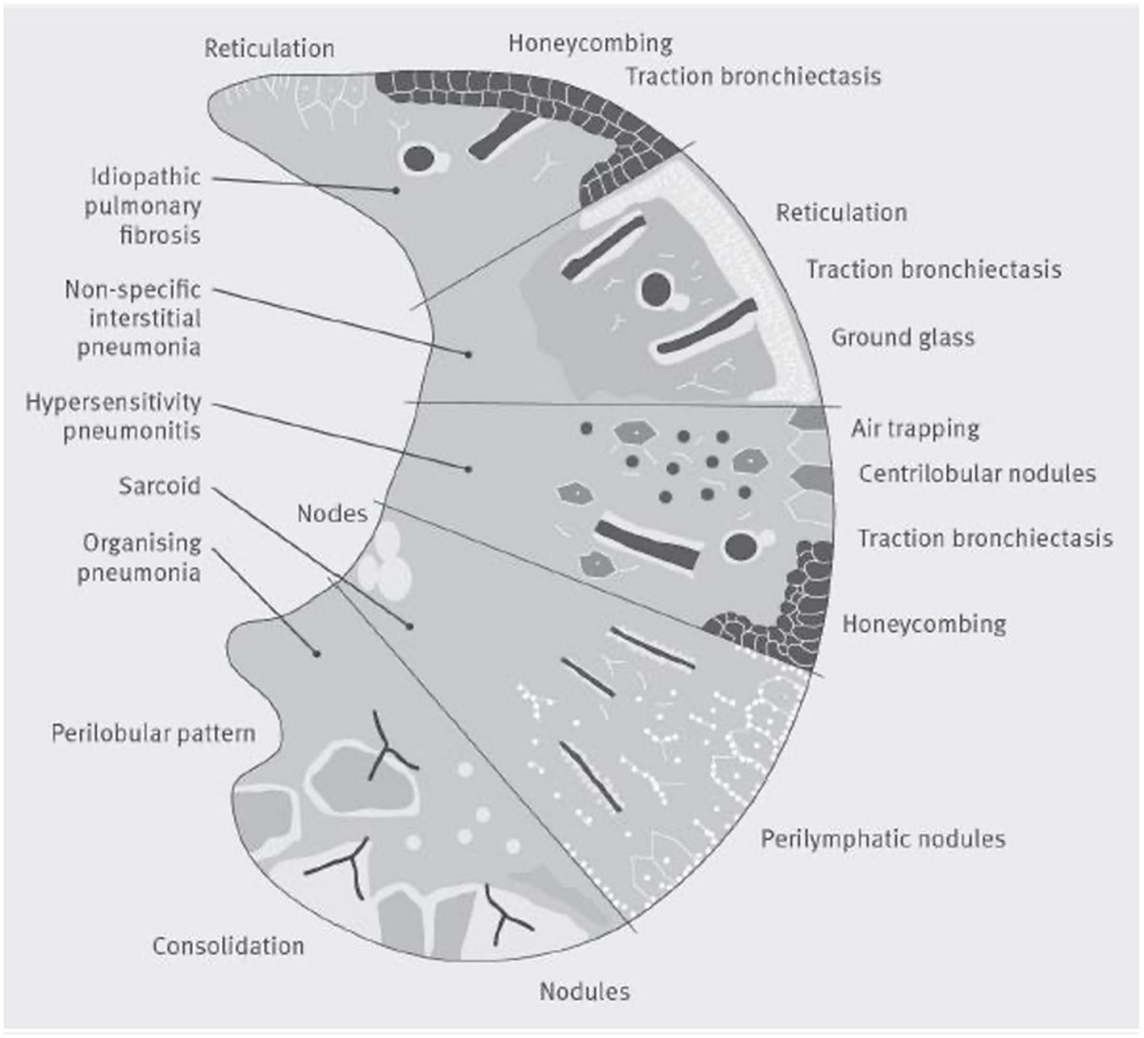
A visual representation of patterns of damage that could be seen on a CT scan in several common interstitial lung diseases. The lower section of the figure represents organizing pneumonia, where a CT may show consolidaiton, a perilobular pattern and nodules. The second section shows Sarcoidosis, where a CT often shows bilateral symmetrical hilar lymph nodal enlargment, fibrosis in the posterior segments of the upper lobes and perilymphatic nodules. The middle section represents hypersensitivity pneumonitis, where focal ground-glass opacity, air trapping, increased attenuation lung in the lung forming the three-density sign can be seen with or without reticulation, traction bronchiectasis and honeycomb cysts. The section second from top illustrates nonspecific interstitial pneumonia, whilst the top section shows a usual interstitial pneumonia pattern which if basal and lower zone predominant in the absence of an identifiable cause of disease could be diagnosed by a multidisciplinary team as idiopathic pulmonary fibrosis.

A comprehensive investigation of radiological imaging should be provided by a specialized thoracic radiologist who is part of the multidisciplinary team of ILD diagnosis (100). Basal predominant traction bronchiectasis or honeycombing in the absence of features suggesting alternative diagnoses suggest a usual interstitial pneumonia pattern on CT. In the presence of no identifiable cause of disease, these cases can be diagnosed as idiopathic pulmonary fibrosis by a multidisciplinary team (Figure 5) (99).

### Invasive testing

Healthcare providers have access to a range of invasive options, such as bronchoscopy lavage (BAL), transbronchial biopsy (TBBX), and surgical lung biopsy (SLB) to obtain definitive histopathologic evidence for various lung diseases (4). Bronchoscopy is an endoscopic procedure designed to visualize the airways. It has both diagnostic and therapeutic applications. For instance, during bronchoscopy, bronchoalveolar lavage (BAL), biopsies, cytology, transbronchial needle aspiration, and endobronchial ultrasound can be performed for diagnostic purposes. On the therapeutic front, it allows interventions such as balloon dilatation, ablation, brachytherapy, and stent placement (4). Bronchoalveolar lavage (BAL) plays an especially significant role in diagnosing diffuse lung diseases (112). The procedure involves instilling a saline solution into a segment of the lung and then collecting the fluid for analysis. The BAL fluid's differential cell counts, microbiologic studies, and cytology contribute to diagnosing various diseases. Particularly in the context of Interstitial Lung Disease (ILD), cellular analysis of the BAL fluid can aid in identifying the underlying cause and potentially excluding IPF (9). If the diagnosis remains elusive even after the bronchoscopy with BAL, a surgical lung biopsy may then be deemed appropriate. This procedure involves the removal of a small piece of lung tissue for laboratory examination and testing. This can provide definitive histopathologic evidence of the disease process (9). However, latest guidelines suggest transbronchial lung cryobiopsy (TBLC) could be a preferable option to surgical lung biopsy in well-equipped centers, due to its association with fewer adverse events (5, 103, 113, 114). Biopsies can be obtained via several methods, such as video-assisted thoracoscopic surgery (VATS) or open lung biopsy. However, these are more invasive procedures with inherent risks, usually reserved for cases where non-invasive techniques and bronchoscopy with BAL fail to yield a definitive diagnosis. These procedures may have complications, including pneumothorax and postoperative pneumonia. A biopsy would aid in diagnosis, but it might be impractical due to patient comorbidities or refusal. In these scenarios, multidisciplinary team discussions play a crucial role in establishing the most probable “working diagnosis,” integrating all available information, including demographic data, disease behavior pre and post-treatment, and bronchoalveolar lavage findings.

## Interstitial lung disease management

**Figure 6:**
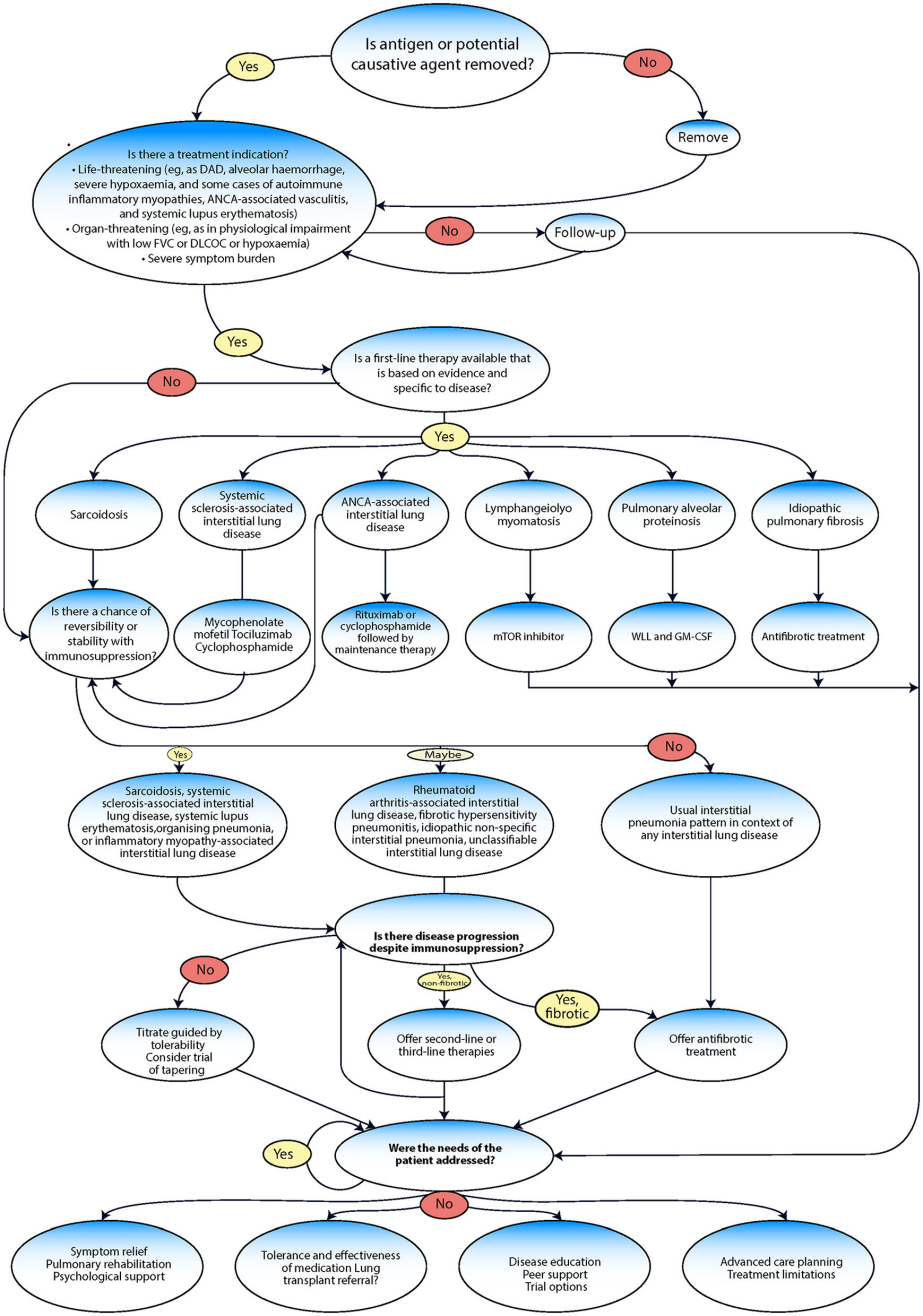
Management algorithm adapted from Wijsenbeek et al. (9).

The management of ILD often follows a series of evaluation steps and treatment pathways (Figure 6) (99, 115). Efforts should be made to provide symptom relief, which could involve various strategies, such as pulmonary rehabilitation (PR) and psychological support, to alleviate symptoms and improve the overall wellbeing of the patient. After providing symptom relief, the next crucial step involves assessing the patient's tolerance to and the effectiveness of any medication being used (116). In severe cases where medications are not effective, a lung transplant referral may be considered. Disease education and peer support can be crucial. Patients should be made aware of their condition, its potential implications, and how to manage it effectively. Peer support can also help the patient cope better with the disease. The management of ILD includes pharmacological treatment approved by the FDA, such as nintedanib and pirfenidone, management drugs such as oral corticosteroids (including prednisone), and others, such as mycophenolate mofetil (CellCept^®^), azathioprine (Imuran^®^), and cyclophosphamide (Cytoxan^®^) (117, 118). Non-pharmacological management includes ceasing medications known to cause ILD; avoiding occupational and environmental exposure to toxins and antigens; having vaccinations (including annual influenza vaccination); quitting smoking; and receiving treatment with PR, supplemental oxygen, and lung transplantation (10).

### Pharmacological management

ILDs are a complex group of diseases with diverse causes, meaning the most effective treatments will vary from patient to patient and depend on the specifics of their condition (119). For instance, sarcoidosis is managed primarily through immunosuppression (120, 121). Systemic sclerosis-associated (SSc) ILD is treated with mycophenolate mofetil, tocilizumab, and cyclophosphamide. In the case of ANCA-associated ILD, the main treatments are rituximab and cyclophosphamide. Lymphangioleiomyomatosis (LAM) is treated using an inhibitor of the mechanistic target of rapamycin mTOR inhibitor. Pulmonary alveolar proteinosis (PAP) is managed with whole lung lavage (WLL) and granulocyte-macrophage colony-stimulating factor (GM-CSF). Finally, IPF is treated with antifibrotic medications (19).

#### Immune suppression therapies

Drugs that suppress the immune system employ a broad range of mechanisms to reduce the body's immune responses, critical for preventing organ rejection after transplantation or treating autoimmune conditions, such as lupus, psoriasis, and rheumatoid arthritis (117, 122). Widely used immunosuppressants include cyclosporine A, tacrolimus, glucocorticoids, methotrexate, and biological agents (e.g. rituximab). Unfortunately, these drugs can lead to an increased risk of infections and the development of malignancies (117).

Corticosteroids, such as prednisolone, are one of the most commonly used immunosuppressive therapies for ILD (123). They have broad anti-inflammatory and immunosuppressive effects. However, they are associated with significant side effects, such as weight gain, indigestion problems, restlessness, sweating, and mood changes (120). These side effects are particularly notable when the substances are used in high doses or for prolonged periods. Thus, corticosteroids often are employed in conjunction with other drugs to reduce the dose needed and mitigate side effects. Other immunosuppressants that have been used in the treatment of ILD include mycophenolate mofetil and azathioprine (124). These drugs are typically prescribed to organ transplant patients, but they have also shown some efficacy in treating ILD. Rituximab, a monoclonal antibody that depletes B cells, is another agent which has been studied for use in ILD, especially in patients who fail to respond to traditional immunosuppressive therapies (117, 125).

Patients historically relied on either antifibrotic medication for IPF or immunosuppressive treatments for non-IPF ILD. Antifibrotic agents, including pirfenidone and nintedanib, have been approved for use in IPF, with nintedanib also approved for fibrotic ILDs with progressive phenotypes, such as progressive pulmonary fibrosis (PFF) (7, 8). Preclinical investigations suggest such antifibrotic drugs could be effective in managing pulmonary fibrosis resulting from a variety of causes. Moreover, they are reported to possess anti-inflammatory properties (7, 8).

Nintedanib is an intracellular tyrosine kinase inhibitor (TKI) demonstrated to have antifibrotic properties (7, 8). The SENSCIS trial (126) was the first phase III study to evaluate the efficacy and safety of nintedanib in patients with non-IPF ILD. Following the results of this trial, the FDA granted approval for nintedanib to be used for the treatment of systemic sclerosis (SSc) ILD (7, 126). The INBUILD trial (127) that led to the approval of this drugs for treatment of any progressive pulmonary fibrosis (PFF) documented an ~50% decrease in the rate of forced vital capacity (FVC) decline in the treated group relative to the placebo group. Such treatment can also potentially mitigate the risk of exacerbation and death. Finally, the INPULSIS-1 and INPULSIS-2 studies were 52-week, randomized, and double-blind phase 3 trials designed to examine the efficacy and safety of nintedanib in patients with IPF (7, 128). These trials found a significant FVC rate decline in those who received a placebo compared to those who received nintedanib.

Several studies of pirfenidone (17, 129–131) that included patients with a variety of ILD subtypes, who had not responded to traditional treatment, also showed encouraging safety profiles and the potential efficacy of this drug. Antifibrotic medications, such as nintedanib, have shown considerable potential in treating both IPF and non-IPF ILDs. Their safety profiles, along with the potential they show in slowing FVC decline and reducing other complications, make them an important area of study for further clinical trials.

### Non-pharmacological management

A range of non-pharmacological methods can be employed to address various aspects of fibrotic ILD regardless of disease stage or cause. Patients with ILD should receive regular influenza and pneumonia vaccines with their treatments as a preventive measure for seasonal infections (4). It is also important for patients to stop smoking, avoid harmful substances, protect themselves from harmful work conditions, and discontinue any drugs that might lead to ILD. Due to frequent physical deterioration in severe cases, a pulmonary rehabilitation program designed for lung patients could potentially reduce their symptoms and improve their physical abilities (14, 132). Similarly, long-term oxygen therapy (133) can be helpful for some. Comorbid conditions and overall physical frailty can significantly decrease quality of life; however, addressing these through screenings and appropriate management can improve a patient's situation (133). Those with a severe form of ILD, known as PF ILD, may be eligible for a lung transplant. For severe ILD patients who cannot undergo transplantation due to age >65 years, class I obesity (BMI 30–34.9 kg/m^2^), severe malnutrition, or other factors, managing symptoms and improving comfort should be the main goal (134, 135).

#### Symptom management

Patients with ILD have different symptoms prognoses. Some can live many years with a disease that responds well to treatment, but others, particularly those with progressive idiopathic fibrotic ILD (PIF-ILD), have a shorter life expectancy similar to that of lung cancer patients (136). Regardless of their specific condition, ILD patients usually have common symptoms that significantly affect their quality of life, including breathlessness, cough, heartburn, and depression (2, 118). These patients often also have sleep issues, feel fatigued, lose weight, and suffer from a loss of appetite. Managing these symptoms can be challenging due to the psychological stress of living with a chronic, life-limiting disease. Hence, a combined approach that includes patient education and self-management is crucial. Several drug and non-drug therapies are available to alleviate symptoms in patients with IPF. These include mild narcotics, respiratory rehabilitation exercises (e.g. pursed-lip breathing), and supplemental oxygen.

#### Pulmonary rehabilitation

Pulmonary rehabilitation (PR) is a comprehensive intervention for patients with chronic respiratory diseases who are symptomatic and often have decreased daily life activities, as recently defined by the European Respiratory Society (ERS) and American Thoracic Society (ATS) (137). The first official definition of PR was published by ATS, which described it as the “an art of medical practice wherein an individually tailored, multidisciplinary program is formulated which through accurate diagnosis, therapy, emotional support and education, stabilizes or reverses both the physio-and psychopathology of pulmonary diseases and attempts to return the patient to the highest possible capacity allowed by his pulmonary handicap and overall life situation” (138). PR offers many benefits for patients with ILD, including improved physiological condition, symptom relief, better psychological health, and cost savings. Past studies have shown positive outcomes for those undergoing PR, such as an improvement in both the 6-min walking test (6 MW) distance, exercise capacity, and overall quality of life (137).

ILD patients may also benefit from supplemental oxygen therapy being a common treatment for ILD (139). It addresses low oxygen levels in the blood and can stop or slow down the development of problems such as high blood pressure in the lungs, heart-related issues, or cognitive dysfunction. How much and how often oxygen is prescribed can vary greatly among ILD patients. Nevertheless, being able to move around, work, exercise, and even travel while using oxygen can significantly improve a patient's life (140).

#### Lung transplantation

Lung transplantation remains the best option for patients with advanced IPF, with ~66% of transplant recipients living for more than 3 years after the surgery and 53% surviving for over 5 years (139). The criteria for lung transplantation in ILD patients encompass UIP or NSIP evidence, a 10% FVC or 15% DLCO decrease over 6 months, an oxygen saturation <88%, a 6-min walk of fewer than 250 m, a 50-m decrease in 6MWD within 6 months, or pulmonary hypertension. The evaluation and waiting period for lung transplantation can last for years, making it a challenging and difficult time for patients and their families (141). While post-transplantation survival rates in IPF patients are around 66% beyond 3 years and 53% beyond 5 years (142), lung transplantation can be a life-saving procedure that significantly improves life expectancy and enhances quality of life for some patients with IPF (135, 143). When lung transplantation is being considered, it is crucial to review the patient at a specialist center. Transplant medicine experts can thoroughly assess the patient's suitability for this major surgical procedure, help prepare the patient, and manage their post-transplant care. The shared care approach is continued in these cases to ensure the best possible patient outcome (100).

#### Palliative care

ILDs can greatly affect a patient's functional abilities, which can cause physical and psychological distress as the disease worsens (144). The WHO defines such care as “an approach that improves the quality of life of patients and their families through the prevention and relief of suffering by means of early identification and impeccable assessment and treatment of pain and other problems, physical, psychosocial and spiritual” (145). Palliative care, which focuses on managing the discomfort, symptoms, and stress of severe illness, should be started as soon as ILD is diagnosed. The main objective of palliative care is to relieve suffering and help the patient and their caregivers live as well as possible. It should be provided in conjunction with other treatments and should involve both primary caregivers and specialist teams (146). Although palliative care is an essential part of a good healthcare approach for patients with organ failure, end-stage chronic illness, issues related to aging, cancer, and cardiovascular diseases, only 14% of patients receive it, according to the World Health Organization (WHO) (145).

Palliative care has been found, for example, to improve survival in patients with cancer (147). Although a poor understanding of palliative care services had previously been reported among clinicians (148), palliative care teams are now available with well-trained and specialized providers (149). Palliative care takes a holistic view of patient care, focusing on these key areas to provide comprehensive support, specifically, relieving discomfort and other challenging symptoms considering both the psychological and spiritual aspects of patient care, improving the overall quality of life and illness progression. Care can be implemented at any stage of illness, alongside other life-prolonging treatments, such as chemotherapy or radiation therapy, and includes necessary diagnostic procedures to manage symptoms effectively. It offers support to help patients live as fully as possible until death, acknowledges death as a natural part of life; employs a team-based approach to meet the needs of both patients and their families, and includes grief counseling when necessary.

New guidelines (146) emphasize the importance of palliative care for patients with ILD. However, palliative care services are still limited and not well implemented. A recent systematic review reported that only about 38% of ILD patients were referred to palliative care. Barriers included fear of discussing the disease trajectory (150, 151) and limited awareness about the actual role of palliative care among patients (148, 152). Hospice care should be considered when a patient is not expected to live more than 6 months. Given the unpredictable nature of ILD, especially IPF.

## Prognosis

The prognosis and severity of ILD depend on the type of ILD and factors such as comorbidities, rate of lung function decline, smoking status, age and the individual's overall health (153). The outcome and course of interstitial lung diseases, including recovery, potential complications, and duration, vary greatly among patients. The classification of the disease, severity, and demographic play a crucial role in predicting its future. It is also necessary to manage comorbid diseases, which can significantly affect survival rates in patients with ILD (154). For example, in patients with IPF, death for non-respiratory reasons was associated mainly with cancer, heart failure, stroke, and coronary artery disease. Recently, a systematic review with a meta-analysis examining the prognosis of IPF reported survival rates of 88% at 1–2 years and 31% at >5 years among patients diagnosed with IPF (155).

To better understand the outcome and course of ILDs, it is essential to assess key physiological parameters and functional indicators. For idiopathic lung disease patients, multiple factors have been shown to be indicators of a poor prognosis and negatively impact the quality of life. These include:

➢ A decrease in 6-min walk (6 MW) distance by more than 150 m within 4 year.
➢ A decline in forced vital capacity (FVC) by more than 10% within 6 months.
➢ A reduction in diffusing capacity for carbon monoxide (DLCO) by more than 15% within 6 months.

## Progression

Understanding disease progression is crucial in progressive pulmonary fibrosis (PPF) and fibrosing ILD as it impacts patient management and treatment strategies (124). PFF refers to a specific form of ILD (5). It applies to a patient with an ILD of known or unknown etiology who exhibits radiological evidence of pulmonary fibrosis, but they do not meet the criteria for idiopathic pulmonary fibrosis (IPF) (5, 124).

Progressive fibrosing ILDs affect 2.2–20.0 out of every 100,000 people in Europe and 28.0 out of 100,000 in the USA (156). Furthermore, a range of 18%−32% of ILDs are estimated to develop into this progressive fibrosing state (116).

The definition of disease progression in patients with fibrosing ILD lacks uniformity (9, 13, 17, 124). Clinical trials have attempted to establish criteria for the identification of individuals whose non-idiopathic pulmonary fibrosis is progressing.

Clinical trials, such as the INBUILD trial, often provide more specific criteria for defining disease progression (127). The criteria used in the INBUILD trial include a relative decline in FVC of 10% or more, a relative decline in FVC of between 5 and 10% coupled with increased fibrosis on high-resolution computed tomography (HRCT), a relative decline in FVC of between 5% and <10% coupled with worsening respiratory symptoms or worsened respiratory symptoms and increased fibrosis on HRCT only. This and similar trials provide an empirical framework for assessing disease progression, which can influence both patient management and the development of new therapeutic strategies.

Another notable study is the ILD trial, which evaluates unclassifiable ILDs. This trial defines disease progression as an absolute decline in FVC of more than 5% or significant symptomatic worsening which is not as a result of cardiac, pulmonary (except worsening of underlying uILD), vascular or other causes. The uILD trial highlights the importance of distinguishing disease progression from other comorbid conditions or secondary complications, especially when the ILD is unclassifiable (157).

The RELIEF study offers another criterion for assessing progression in fibrosing ILDs. This trial uses a slope calculation based on at least three values, documenting an annualized decline in FVC of 5% (absolute) or more despite appropriate conventional therapy. This approach stresses the progressive nature of these diseases as it demonstrates how patients may still experience a significant deterioration of their lung function, notwithstanding optimal standard treatment (158).

Kreuter et al. described disease progression as a composite of at least a 10% decline in FVC, a 50-m decline in the 6-min walk distance (6MWD), or death (159). In addition, ILD progression has been defined as a decline in lung function, worsened respiratory symptoms, and unresponsiveness to anti-inflammatory or immunomodulatory treatments (24).

The outcomes of these trials have helped lead to the development of a universally accepted definition for PPF, which is outlined below. These parameters provide clinicians with a structured approach to identifying and managing disease progression in patients with fibrosing ILDs, despite the inherent challenges associated due to the diverse nature of these conditions (124). The criteria for identifying PPF requires at least two of the following three symptoms within the past year, given that no other explanation for these symptoms is found (5, 9):

1. Worsening respiratory symptoms: these could include increased shortness of breath, worsening cough, or other symptoms associated with deteriorating lung function.
2. Physiological evidence of disease progression can be determined by either of the following: (a) An absolute decline in forced vital capacity (FVC) >5% predicted within a year of follow-up. (b) An absolute decline in the diffusing capacity of the lung for carbon monoxide (DLCO, corrected for hemoglobin) >10% predicted within a year of follow-up.
3. Radiological evidence of disease progression, as indicated by one or more of the following: (a) Increased extent or severity of traction bronchiectasis and bronchiectasis. (b) New ground-glass opacity with traction bronchiectasis. (c) Emergence of fine reticulation. (d) The increased extent or increased coarseness of reticular abnormality. (e) New or increased honeycombing. Increased lobar volume loss (5).

While these criteria offer a structured approach to defining disease progression in fibrosing ILDs, it is important to note that predicting the course of disease for an individual patient remains a significant challenge. Factors such as decline in FVC, lower DLCO, honeycombing on HRCT and scoring systems used can be indicative of disease progression in ILDs characterized by PPF (5). Despite current limitations, these evolving approaches represent significant progress toward personalized medicine in the management of PF-ILDs.

In summary, research has established a significant association between FVC decline in IPF patients and increased mortality rates, particularly in cases with an absolute FVC decline of 10%−15% (160). However, validated biomarkers for disease progression are still lacking (56), and the irregular measurement of Forced Vital Capacity (FVC) remains a common practice (161).

## Acute exacerbation in ILD

Acute exacerbation in patients with ILD was first defined by Kondoh: “(1) exacerbation of dyspnea within a few weeks; (2) newly developing diffuse pulmonary infiltrates on chest x-ray films; (3) deterioration of hypoxemia (PaO_2_/fractional concentration of oxygen in inspired gas FIO_2_ <225); and (4) absence of apparent infectious agents” (162). Collard et al. (163) defined an exacerbation as “acute, clinically significant respiratory deterioration characterized by evidence of new widespread alveolar abnormality, typically less than 1-mo's duration with the exclusion of alternative etiologies.” It was also defined by Raghu et al. (5) as an acute clinically significant deterioration of respiratory symptoms without an identified cause sudden worsening of respiratory symptoms that lasts less than a month. Computed tomography scans typically show the presence of a new ground-glass opacity and/or consolidation and the absence of heart failure or fluid overload.

The National Institutes of Health-sponsored IPF Clinical Trials Network (IPFnet) proposed the diagnostic criteria of acute exacerbation and described the clinical, radiological and histopathological presentation of patients with IPF (163). The following criteria were proposed for diagnosing an acute exacerbation in IPF:

➢ A worsening of clinical symptoms lasting <30 days.
➢ The appearance of new bilateral ground-glass opacification and/or consolidation on high-resolution computed tomography (CT).
➢ Deterioration not fully explained by cardiac failure or fluid overload the absence of alternative etiologies, such as heart failure, or pulmonary embolism.

The incidence of acute exacerbation of idiopathic pulmonary fibrosis (AE-IPF) varies depending on the study cohort, with a range from 2% to 15% per year (164, 165). However, the prevalence of AEs is lower in non-IPF ILD (166), with an average of 2% per year. This difference in incidence is likely due to the different underlying pathologies of AE-IPF and non-IPF ILD. AE-IPF is characterized by progressive fibrosis of the lung tissue, while non-IPF ILD is characterized by a variety of other pathologies, such as inflammation, infection, and autoimmune disorders.

The diagnosis process for acute exacerbation in ILD commences with a thorough history and physical examination. If this initial step identifies an extra-pulmonary diagnosis, then it is not an acute exacerbation. Conversely, when no extra-pulmonary condition is detected, the timeframe of respiratory deterioration is assessed. If the worsening lasts beyond a month, it is not an acute exacerbation. However, if the respiratory decline has occurred within a month, the subsequent step is a CT scan of the chest. If this scan reveals alternative conditions such as pneumothorax, pleural effusion, or pulmonary embolism, the diagnosis would again rule out acute exacerbation. In the absence of these alternatives, the existence of new bilateral ground-glass opacities (GGO) or consolidation becomes the critical element. If such features are missing, acute exacerbation is ruled out. Should the CT scan indicate new bilateral GGO or consolidation, then possible causes such as cardiac failure or fluid overload are considered. If such conditions can account for the CT findings, it is not an acute exacerbation. However, if these findings remain unexplained, the diagnosis is an acute exacerbation of ILD. The final determination involves identifying possible triggers for the exacerbation, such as infection or post-procedural/operative complications. If a trigger is found, it qualifies as a triggered acute exacerbation, whereas the lack of a recognizable trigger would lead to a diagnosis of idiopathic acute exacerbation (163).

Acute exacerbation of interstitial lung disease (AE-ILD) can arise from various forms of ILD, but a higher risk is associated with usual interstitial pneumonia (UIP)-like lesions in chronic hypersensitivity pneumonitis (HP) and in patients with connective tissue disease-interstitial lung disease (CTD-ILD). Other risk factors include male gender, concurrent pulmonary hypertension, coronary artery disease, increased body mass index, and increased exposure to ozone and nitrogen dioxide. Inconsistencies exist regarding former smoking and age as risk factors. A retrospective study revealed that ~22% of ILD and lung cancer patients undergoing chemotherapy experienced AE-ILD, suggesting certain chemotherapy agents as safer options. Surgical biopsy is another significant risk factor for AE-ILD, with incidence rates varying from <2.5% after ILD diagnosis biopsy to 3%−32% post-pulmonary resection for lung cancer. Lower FVC and diffusing capacity for carbon monoxide serve as additional risk factors for respiratory decline post-surgery. AE-ILD incidence has also been reported in non-pulmonary major surgeries, and a potential association between AE-ILD and bronchoalveolar lavage (BAL) is observed in IPF. Future research needs to explore the risk of AE-ILD post-cryobiopsy, given the currently limited data (159).

AE-ILD is a severe condition, leading to high mortality rates; between 35 and 46% of IPF-related deaths arise from AE-IPF. Mortality rates during the hospital stay for AE-IPF patients can exceed 50%, and the median survival period ranges from 1 to 4 months post-AE-IPF. One-month mortality varies from 37 to 53%, while 3-month mortality is between 63.8% and 73.7%. IPF patients have worse survival rates than other ILD sufferers, but AE-ILD is fatal across all ILD types. Prognostic factors include diminished baseline pulmonary function and oxygenation, advanced fibrosis or disease on HRCT, >15% lymphocytosis in BAL, and several blood markers.

Treatment of acute exacerbations of interstitial lung disease (AE-ILD) typically involves high-dose corticosteroids and antibiotics, but their efficacy is not supported by solid evidence. Guidelines recommend supportive care such a symptom palliation and oxygen supply and discourage mechanical ventilation due to the high mortality. Various treatment regimens, including steroid therapy with tacrolimus or cyclosporine, rituximab with plasma exchange and intravenous immunoglobulin, and intra-venous thrombomodulin, have shown potential benefits. Anti-acid therapy and antifibrotic drugs could potentially prevent AE-IPF, though further data is needed. A non-steroid approach with the best supportive care and broad-spectrum antibiotics shows varying survival outcomes, depending on previous treatments.

Obtaining bronchoalveolar lavage (BAL) biomarkers and conducting HRCT can prove to be a significant challenge. However, the advent of daily remote monitoring, especially home spirometry, offers a potential, non-invasive solution (167). These tools provide the means to safely identify patients who are at increased risk for developing AE-ILD, promote a deeper understanding of AE-PF clinical progression, and potentially enable early detection of AE-IPF. Given the supportive evidence that shows how home spirometry can enhance clinical trial outcomes for IPF therapeutics, we should direct our focus toward studies assessing the benefits of daily home spirometry for ILD patients (168). This focus leads us seamlessly into the broader application of remote monitoring. Using questionnaire responses to monitor symptoms like fatigue, cough, anxiety, depression, and quality of life, as well as tracking vital physiological parameters such as heart rate, oxygen saturation, respiratory rate, and FVC; we can gain a comprehensive view of a patient's state which sustains a practical approach to disease management.

## Author contributions

MA: Conceptualization, Data curation, Formal analysis, Investigation, Methodology, Project administration, Software, Supervision, Validation, Visualization, Writing – original draft, Writing – review & editing. A-MR: Conceptualization, Investigation, Methodology, Project administration, Supervision, Validation, Visualization, Writing – original draft, Writing – review & editing. JJ: Conceptualization, Project administration, Supervision, Validation, Visualization, Writing – review & editing. YR: Conceptualization, Project administration, Supervision, Validation, Visualization, Writing – original draft, Writing – review & editing. AF: Conceptualization, Data curation, Investigation, Methodology, Project administration, Supervision, Validation, Visualization, Writing – original draft, Writing – review & editing. JH: Data curation, Formal analysis, Methodology, Project administration, Supervision, Validation, Conceptualization, Investigation, Visualization, Writing – original draft, Writing – review & editing. JP: Conceptualization, Data curation, Formal analysis, Investigation, Methodology, Project administration, Supervision, Validation, Visualization, Writing – original draft, Writing – review & editing.
